# European Surveillance for West Nile Virus in Mosquito Populations

**DOI:** 10.3390/ijerph10104869

**Published:** 2013-10-11

**Authors:** Olivier Engler, Giovanni Savini, Anna Papa, Jordi Figuerola, Martin H. Groschup, Helge Kampen, Jolyon Medlock, Alexander Vaux, Anthony J. Wilson, Doreen Werner, Hanna Jöst, Maria Goffredo, Gioia Capelli, Valentina Federici, Mauro Tonolla, Nicola Patocchi, Eleonora Flacio, Jasmine Portmann, Anya Rossi-Pedruzzi, Spiros Mourelatos, Santiago Ruiz, Ana Vázquez, Mattia Calzolari, Paolo Bonilauri, Michele Dottori, Francis Schaffner, Alexander Mathis, Nicholas Johnson

**Affiliations:** 1 Spiez Laboratory, Federal Office for Civil Protection, Austrasse, Spiez 3700, Switzerland; E-Mails: oliver.engler@babs.admin.ch (O.E.); jasmine.portmann@babs.admin.ch (J.P.); 2 Zooprofilactic Institute Abruzzo and Molise “G. Caporale”, Campo Boario, Teramo 64100, Italy; E-Mails: g.savini@izs.it (G.S.); m.goffredo@izs.it (M.G.); v.federici@izs.it (V.F.); 3 Department of Microbiology, Medical School, Aristotle University of Thessaloniki, Thessaloniki 54124, Greece; E-Mail: annap@med.auth.gr; 4 Department of Wetland Ecology, Estación Biológica de Doñana, CSIC, Avda. Américo Vespucio s/n, Sevilla 41092, Spain; E-Mail: jordi@ebd.csic.es; 5 Friedrich-Loeffler-Institute, Federal Research Institute for Animal Health, Greifswald—Insel Riems, Südufer 17493, Germany; E-Mails: martin.groschup@fli.bund.de (M.H.G.); helge.kampen@fli.bund.de (H.K.); 6 Public Health England, Medical Entomology group, MRA, Emergency Response Department, Porton Down, Salisbury SP4 0JG, UK; E-Mails: jolyon.medlock@phe.gov.uk (J.M.); alex.vaux@phe.gov.uk (A.V.); 7 The Pirbright Institute, Ash Road, Pirbright GU24 0NF, UK; E-Mail: anthony.wilson@pirbright.ac.uk; 8 Institute of Land Use Systems, Leibnitz Centre for Agricultural Lanscape Research (ZALF), Eberswalder Strasse 84, Müncheberg 15374, Germany; E-Mail: doreen.werner@zalf.de; 9 German Centre for Infection Research (DZIF), Partner Site Hamburg-Luebeck-Borstel, Hamburg, Germany and German Mosquito Control Association (KABS), Waldsee and Bernhard-Nocht Institute for Tropical Medicine, Hamburg D-20359, Germany; E-Mail: jonassi@gmx.de; 10 Zooprofilactic Institute Venezie, Viale dell’ Università, 10, Padua, 35020 Legnaro, Italy; E-Mail: gcapelli@izsvenezie.it; 11 Institute of Microbiology, Laboratory of Applied Microbiology, Via Mirasole 22a, Bellinzona CH-6500, Switzerland; E-Mail: mauro.tonolla@unige.ch; 12 Mosquito Working Group, via al Castello, Canobbio CH-6952, Switzerland; E-Mails: fbm@bluewin.ch (N.P.); eleonora.flacio@ti.ch (E.F.); anyarossip@hotmail.com (A.R.-P.); 13 EcoDevelopment SA, Thermi 57001, Greece; E-Mail: info@ecodev.gr; 14 Servicio de Control de Mosquitos, Diputación Provincial de Huelva, Huelva E-21003, Spain; E-Mail: sruiz@diphuelva.org; 15 CNM-Instituto de Salud Carlos III, Majadahonda, Madrid 28220, Spain; E-Mail: a.vazquez@isciii.es; 16 Zooprofilactic Institute Lombardy and Emilia Romagna “B. Ubertini”, Brescia 25124, Italy; E-Mails: mattia.calzolari@izsler.it (M.C.); paolo.bonilauri@izsler.it (P.B.); michele.dottori@izsler.it (M.D.); 17 Institute of Parasitology, National Centre for Vector Entomology, University of Zurich, Winterthurerstr 266a, Zurich 8057, Switzerland; E-Mails: francis.schaffner@uzh.ch (F.S.); alexander.mathis@uzh.ch (A.M.); 18 Animal Health and Veterinary Laboratories Agency, Woodham Lane, Surrey KT15, 3NB, UK

**Keywords:** West Nile virus, mosquito, surveillance, vector, invasive species

## Abstract

A wide range of arthropod-borne viruses threaten both human and animal health either through their presence in Europe or through risk of introduction. Prominent among these is West Nile virus (WNV), primarily an avian virus, which has caused multiple outbreaks associated with human and equine mortality. Endemic outbreaks of West Nile fever have been reported in Italy, Greece, France, Romania, Hungary, Russia and Spain, with further spread expected. Most outbreaks in Western Europe have been due to infection with WNV Lineage 1. In Eastern Europe WNV Lineage 2 has been responsible for human and bird mortality, particularly in Greece, which has experienced extensive outbreaks over three consecutive years. Italy has experienced co-circulation with both virus lineages. The ability to manage this threat in a cost-effective way is dependent on early detection. Targeted surveillance for pathogens within mosquito populations offers the ability to detect viruses prior to their emergence in livestock, equine species or human populations. In addition, it can establish a baseline of mosquito-borne virus activity and allow monitoring of change to this over time. Early detection offers the opportunity to raise disease awareness, initiate vector control and preventative vaccination, now available for horses, and encourage personal protection against mosquito bites. This would have major benefits through financial savings and reduction in equid morbidity/mortality. However, effective surveillance that predicts virus outbreaks is challenged by a range of factors including limited resources, variation in mosquito capture rates (too few or too many), difficulties in mosquito identification, often reliant on specialist entomologists, and the sensitive, rapid detection of viruses in mosquito pools. Surveillance for WNV and other arboviruses within mosquito populations varies between European countries in the extent and focus of the surveillance. This study reviews the current status of WNV in mosquito populations across Europe and how this is informing our understanding of virus epidemiology. Key findings such as detection of virus, presence of vector species and invasive mosquito species are summarized, and some of the difficulties encountered when applying a cost-effective surveillance programme are highlighted.

## 1. Introduction

West Nile virus (WNV) is classified within the family *Flaviviridae* and genus *Flavivirus.* Five WNV lineages have been identified based on genomic phylogeny. Lineage 1 dominates, and contains viruses detected throughout Africa [1], Asia [2] and since 1999, the Americas. Kunjin virus, isolated in Australia [3], is now considered a subtype of WNV Lineage 1. Lineage 2, originally thought to be restricted to sub-Saharan Africa, has been recently detected in Austria, Greece, Hungary, Italy and Russia [4]. Lineage 3 was isolated in the Czech Republic [5], Lineage 4 was isolated in Russia [6] and Lineage 5, isolated in India [7]. A putative novel lineage has been detected in Spain in 2006 [8].

Primary hosts for the virus are birds, which usually do not show clinical signs, but considerable avian mortality has been observed in Israel and North America [9,10]. Numerous other vertebrates can be infected with WNV, with clinical disease primarily affecting horses and humans [11,12]. Mosquitoes are the main biological vectors of WNV; in addition, the virus has been detected in ticks [13], and WNV has been mechanically transmitted in the laboratory by large biting flies but their vector role remains unclear. Seroconversion, often as early as five days post-infection, leads to rapid clearance of virus from the blood following the development of antibodies [14]. Infection in humans is usually asymptomatic or a mild febrile illness, referred to as West Nile fever, while in less than 1% of infections there is involvement of the nervous system (encephalitis, meningitis, acute flaccid paralysis) with approximately 10% fatality [15]. There is no treatment for WNV infection and no vaccine has been approved for human use to date. Equines are the main domestic animals that develop disease following infection with WNV. A minority of infected horses, approximately 10%, develop neurological signs of disease including ataxia, paresis or hind limb paralysis, skin fasiculations, muscle tremors and rigidity [16]. Experimental infection of horses has confirmed that a low level of viremia of short duration develops that is insufficient to allow the horse to act as an amplifying host for WNV [17]. Commercial vaccines are now available for the vaccination of horses based on formalin inactivated virus with adjuvant [18].

The ability of different mosquito species to acquire and transmit WNV is highly variable [19]. The first isolations of WNV were made from *Culex* (*Cx.*) species [20] which are accepted as the primary global transmission vector. In North America a large number of *Culex* species have been shown to be competent for WNV transmission [21]. In Europe, *Cx. pipiens*, *Cx. perexiguus*, and *Cx. modestus* are important vector species [22,23], whereas in Australia, *Cx. annulirostris* is considered the major vector of Kunjin virus [24]. In South Africa *Cx. univittatus* has been shown to efficiently transmit WNV to birds [25]. WNV has been detected in a further ten genera of mosquitoes including *Ochlerotatus* (*Oc*.), *Aedes* (*Ae.*), *Anopheles* (*An.*), *Coquillettidia*, *Aedeomya*, *Mansonia*, *Mimomyia*, *Psorophora*, *Culiseta* (*Cs.*) and *Uranoteania* [26]. These can act as bridge vectors critical to transmission from birds to humans and equines. 

The vectorial capacity (C) of a particular species of mosquito can be expressed in the following equation: C = ma^2^bp^n^/(−lnp) where m the vector density in relation to host density; a the vector’s daily blood-feeding rate on a host species; b is the vector competence, *i.e.*, the proportion of vectors that develop infective pathogen stages; p the vector’s daily survival rate; n the duration in days of the pathogens to reach the saliva (extrinsic incubation period, EIP). Some of these parameters can be derived experimentally from laboratory studies [27]. However, one of the goals of surveillance in mosquitoes is to generate data that can be applied to calculating the vectorial capacity or to find surrogates that can measure this. Key parameters that influence this are the density of a particular species, its host preference and host-vector contact rate, and a range of climatic factors that in turn influence the EIP. Transmission of arthropod-borne viruses such as WNV is dependent on all of these factors.

WNV has been responsible for sporadic outbreaks of disease in countries around the Mediterranean Sea since the 1960s [28]. These have involved infections in humans and/or horses (reviewed by [26]). All outbreaks were reported between July and September. Since 2000, there have been further documented outbreaks in Russia [29], Morocco [30], Portugal [31], Italy [32], Greece [33], Austria [34], Tunisia [35] and Spain [36]. The majority of outbreaks have been attributed to infections with WNV Lineage 1. However, the outbreak in Greece is distinct in that it has been dominated by human infections caused by WNV Lineage 2. A number of outbreaks have been reported over consecutive years in Romania, Italy, Spain and Greece. 

Targeted surveillance for the virus within mosquito populations offers an opportunity to detect virus prior to the emergence of disease in equine species or human populations [37]. Additionally, it can establish a baseline of mosquito-borne virus activity and allow monitoring of change to this over time. This provides a window in which to initiate control methods such as vector control, introduce biosecurity measures for livestock or initiate preventative vaccination. However, effective surveillance that predicts virus outbreaks is challenged by a range of factors including poor knowledge of social and ecological factors that determine vector-host contact rate and facilitate transmission to humans and horses. Practical limitations including restricted financial support, difficulties in mosquito identification due to lack of specialist entomologists and the sensitive, rapid detection of viruses in mosquito pools can also impede effective surveillance. A key area of this is the accurate identification of mosquito species as only certain species in any given area can act effectively as bridge vectors to human or equine populations [38]. This article will provide an overview of the mosquito surveillance for WNV currently underway in a number of European countries.

## 2. Survey by Country

### 2.1. Italy

In Italy, a multi-species national surveillance plan that targeted birds, domestic poultry, horses, mosquitoes and humans was implemented by the Italian government in 2001, following the first outbreak of West Nile disease [39]. The aim of this ongoing plan is to detect early introductions of new viruses and monitor the spread of infection. The national programme includes the serological screening of sentinel horses, sentinel-chickens and backyard poultry flocks and the surveillance on all equine neurological cases, resident captured and wild dead birds, and vectors [40,41]. Ten high risk areas have been selected based on the presence of significant numbers of waterfowl and species of migratory birds. The surveillance plan has been updated annually in line with changes in WNV epidemiology. The entomological surveillance is based on a range of collection sites placed either in the at-risk areas or in areas with virus circulation, and aims to capture and identify possible WNV and Usutu virus (USUV) vector species and determine their abundance and distribution. Different traps and methods are used to collect mosquitoes. CO_2_-CDC light-traps, which operate from sunset to sunrise, are used to collect host-seeking adult female mosquitoes of various species. They are placed approximately 1.5 m from the ground and kept working overnight for two consecutive nights; the BG-Sentinel and Gravid traps which are instead placed on the ground in sites protected from animal attacks and kept working for 48 h, are selective for diurnal species and for gravid female mosquitoes of the *Cx.* genus, respectively; aspiration trapping is used to collect engorged mosquitoes in their resting sites (walls of animal shelters); larval collection is also utilized to improve the monitoring of mosquito species.

The data presented in this study were from the mosquito collections performed between 2008 and 2012 on a monthly basis in areas with active virus circulation and from March to October in surveillance zones (areas considered at risk of virus introduction). In surveillance zones CO_2_-CDC and BG-Sentinel traps were used whereas in areas of active virus circulation, CDC gravid traps and aspiration trapping were also employed. Mosquitoes were identified and pooled (a maximum of 50 individuals/pool) by catch site, collection date, trapping method, species, sex and female status (engorged or unfed), and tested for WNV and USUV by real time RT-PCR. The WNV lineage was later identified using lineage specific RT-PCRs. 

According to Severini *et al*., [42], 64 species of mosquitoes belonging to eight genera are present in Italy. Between 2008 and 2012 the West Nile Disease national surveillance plan captured a total of 78,558 mosquitoes belonging to 33 species and seven genera from 3,313 mosquito collections performed throughout Italy (Table 1). In agreement with previous surveys [41], *Cx. pipiens* and *Oc. caspius* were the most abundant species collected. 

Overall 5,184 pools were sorted and tested for WNV and 3,646 for USUV. WNV was detected in 10 pools which included females of *Cx. pipiens*, *Oc. caspius*, and *Cx. modestus*. USUV was detected in seven pools, consisting of *Cs. annulata*, *Oc. detritus* and *Cx. pipiens*. 

Besides the entomological activities supported by the Ministry of Health at national level, more comprehensive surveillance programs were carried out at the Regional level in WNV affected areas.

**Table 1:** Mosquito species collected in Italy in the period 2008–2012 as part of the West Nile Disease National Surveillance Plan (total collections 3,313).

Mosquito species	2008	2009	2010	2011	2012	Total
*Aedes albopictus*	46	317	1,053	1,449	1,179	4,044
*Aedes* sp.	18		2	1	32	53
*Aedes vexans*	2	3	16	16	40	77
*Anopheles algerensis*			1		0	1
*Anopheles claviger*	1	3	16	16	13	49
*Anopheles hyrcanus*					6	6
*Anopheles maculipennis s.l.*	228	658	755	2,675	661	4,977
*Anopheles plumbeus*	63	8	54	213	2	340
*Anopheles* sp.	12	15	16	9	10	62
*Anopheles superpictus*				3	4	7
*Coquillettidia richiardii*	4	5	10	60	16	95
*Culex brumpti*		1		3	0	4
*Culex hortensis*	1	1	31	38	5	76
*Culex impudicus*	7		1		0	8
*Culex laticinctus*			6		0	6
*Culex mimeticus*	1	1	1		0	3
*Culex modestus*	2	12	2	22	0	38
*Culex pipiens s.l.*	5,004	12,487	6,933	12,635	15,448	52,507
*Culex* sp.	45	135	233	1,426	1,048	2,887
*Culex territans*		1	6		3	10
*Culex theileri*		14	422	142	15	593
*Culex univittatus*		68	126	2	0	196
*Culiseta annulata*	78	25	158	250	132	643
*Culiseta litorea*	18				0	18
*Culiseta longiareolata*	55	56	278	392	112	893
*Culiseta* sp.	4	5		1	0	10
*Culiseta subochrea*				1	0	1
*Ochlerotatus atropalpus*			1		0	1
*Ochlerotatus caspius*	1,561	1,775	1,586	1,673	1,603	8,198
*Ochlerotatus communis*	214	1	48	109	45	417
*Ochlerotatus detritus*	430	10	177		42	659
*Ochlerotatus dorsalis*	1		3		0	4
*Ochlerotatus echinus*	2				0	2
*Ochlerotatus geniculatus*			5	1	6	12
*Ochlerotatus rusticus*	72	1	1	1	0	75
*Ochlerotatus* sp.	280	198	10	644	233	1365
*Ochlerotatus zammiti*	187				0	187
*Uranotaenia unguiculata*	7	6	9	5	7	34
**Total**	**8,343**	**15,806**	**11,960**	**21,787**	**20,662**	**78,558**

In the Emilia-Romagna region, a large-scale mosquito survey was established following the first reported case of WNV in a horse in 2008. CO_2_ baited traps were operated every two weeks and mosquito pools (maximum 200 per pool) were submitted for species specific (USUV and WNV) or genus (*Flavivirus* and *Orthobunyavirus*) specific RT-PCRs. More than one million mosquitoes were tested (1,176,771 specimens grouped in 10,393 pools); most of these (80.4%) were identified as *Cx. pipiens*. WNV was detected in 32 pools of *Cx. pipiens* collected between 2008 and 2010 whereas USUV was detected in 310 pools. Of these, 295 were *Cx. pipiens*, 12 *Ae. albopictus* and three from other mosquito species (*An. maculipennis s.l.* and *Oc. caspius*) [37]. Two further orthobunyaviruses, Tahyna and Batai viruses, were also detected during this surveillance period [43]. In Lombardia Region (“Parco Lombardo delle Valli dell Ticino”), a total of 76,922 mosquitoes were collected and grouped into 746 pools. When tested for Flavivirus and Orthobunyavirus, USUV (in five pools of *Cx. pipiens*), Batai and Tahyna virus RNAs were also detected.

In the north-eastern part of Italy comprehensive mosquito monitoring was introduced in Veneto in 2009 and in Friuli Venezia Giulia (FVG) from 2011. CO_2_-CDCs light traps have been activated fortnightly from May to October. A total of 356,926 mosquitoes were collected. Most (86%) were identified as *Cx. pipiens*. Of the 8,393 pools tested, WNV was detected in 29 pools of *Cx. pipiens* (10 in 2010, six in 2011 and 13 in 2012), while USUV in 86 pools of *Cx pipiens* and one of *Oc. caspius* (six in 2009; 23 in 2010, 24 in 2011 and 34 in 2012).

**Figure 1:**
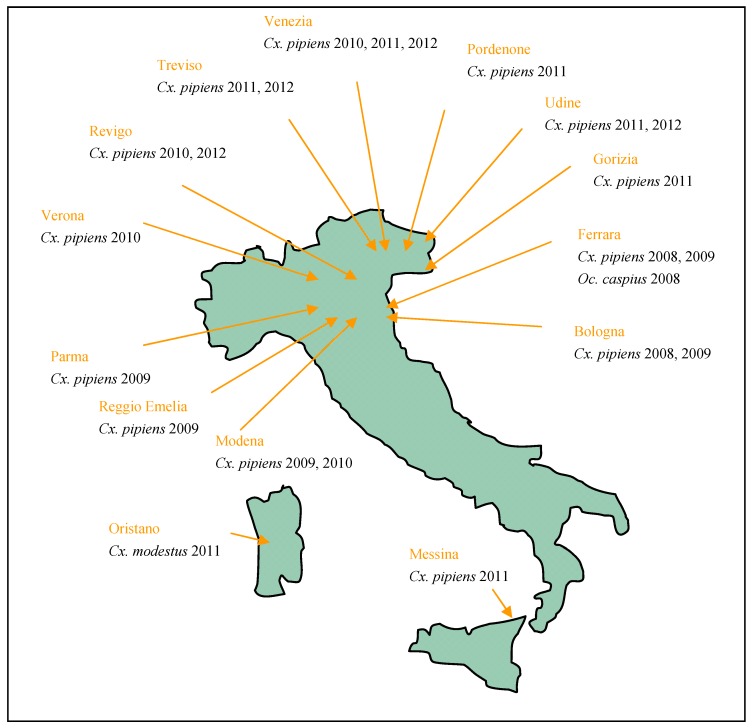
Map showing areas where WNV infected mosquitoes have been trapped in Italy according to the Italian entomological surveillance plan for West Nile disease.

The results of WNV and USUV surveillance in Italian mosquitoes are displayed in Table 2 and Table 3, respectively. WNV RNA was found in mosquitoes collected in Emilia Romagna, Lombardia, Friuli Venezia Giulia, Veneto, Sardinia and Sicily regions, whereas USUV RNA was found in pools from Molise, Veneto, Umbria, Sardinia, Marche and Tuscany regions (Figure 1). Presence of WNV RNA was generally found in pools collected between July and September which is much earlier than the occurrence of human and horse clinical cases. Virus amplification through cycling between resident birds and mosquitoes prior to spillover into other species could account for this temporal discrepancy. WNV Lineage 2 was first detected in Italy in 2011 in birds and pools of *Cx. pipiens* collected in FVG and Veneto regions. It has subsequently been detected in Sardinia in 2012 and in the Veneto region [44,45].

**Table 2:** West Nile virus detected in mosquitoes collected in Italy during national and regional surveillance activities (2008–2012).

Mosquito species	Number of positive/tested pools					
	2008	2009	2010	2011	2012	Total
*Aedes albopictus*	0/96	0/183	0/230	0/689	0/511	0/1,709
*Aedes koreicus*					0/3	0/3
*Aedes* sp.	0/19	0/35	0/1	0/5	0/11	0/71
*Aedes vexans*	0/73	0/122	0/297	0/301	0/204	0/997
*Anopheles claviger*			0/6	0/16	0/10	0/32
*Anopheles hyrcanus*					0/2	0/2
*Anopheles maculipennis s.l.*	0/52	0/113	0/147	0/466	0/239	0/1,017
*Anopheles plumbeus*	0/2	0/16	0/32	0/83	0/23	0/156
*Anopheles* sp.	0/4		0/3	0/4	0/4	0/15
*Anopheles superpictus*					0/1	0/1
*Coquillettidia richiardii*	0/0	0/1	0/4	0/59	0/15	0/79
*Culex brumpti*				0/2		0/2
*Culex hortensis*	0/1		0/2	0/10	0/3	0/16
*Culex impudicus*	0/2		0/1			0/3
*Culex mimeticus*	0/1					0/1
*Culex modestus*	0/13	0/95	0/55	1/57	0/64	1/284
*Culex pipiens*	5/510	27/1,898	13/5,539	8/4,568	13/3,357	66/15,872
*Culex* sp.	0/27	0/3	0/14	0/132	0/147	0/323
*Culex territans*				0/4	0/6	0/10
*Culex theileri*			0/17	0/30	0/11	0/58
*Culex univittatus*			0/46	0/2		0/48
*Culiseta annulata*	0/10	0/18	0/48	0/134	0/62	0/272
*Culiseta longiareolata*	0/1		0/16	0/107	0/48	0/172
*Ochlerotatus geniculatus*	0/16	0/7	0/9	0/8	0/2	0/36
*Ochlerotatus punctor*			0/1			0/1
*Ochlerotatus rusticus*			0/1	0/1		0/2
*Ochlerotatus* sp.		0/2	0/1	0/37	0/45	0/85
*Uranotaenia unguiculata*					0/2	0/2
**Total**	**9/1,222**	**27/3,050**	**13/7,219**	**9/7,768**	**13/5,609**	**71/24,852**

**Table 3:** Usutu virus detected in mosquitoes collected in Italy during national and regional surveillance activities (2008–2012).

Mosquito species	Number of positive/tested pools				
	2009	2010	2011	2012	Total
*Aedes albopictus*	2/175	2/144	6/675	52/12	12/1,506
*Aedes* sp.	0/35	0/0	0/5	0/11	0/51
*Aedes koreicus*				0/3	0/3
*Aedes vexans*	122	0/288	0/301	0/204	0/915
*Anopheles claviger*	0/0	0/0	0/15	0/10	0/25
*Anopheles hyrcanus*	0/0	0/0	0/0	0/2	0/2
*Anopheles maculipennis s.l.*	0/99	0/55	1/442	238	1/834
*Anopheles plumbeus*	0/16	0/6	0/78	0/21	0/121
*Anopheles* sp.	0/0	0/1	0/4	0/4	0/9
*Anopheles superpictus*	0/0	0/0	0/0	0/1	0/1
*Coquillettidia richiardii*	0/1	0/4	0/56	0/15	0/76
*Culex brumpti*	0/0	0/0	0/2	0/0	0/2
*Culex hortensis*	0/0	0/0	0/8	0/3	0/11
*Culex modestus*	0/95	0/53	0/52	0/64	0/264
*Culex pipiens*	63/1,836	112/5,138	105/4,442	112/3,356	392/14,772
*Culex* sp.	0/3	0/1	0/125	1/146	1/275
*Culex territans*	0/0	0/0	0/4	0/6	0/10
*Culex theileri*	0/0	0/4	0/23	0/11	0/38
*Culex univittatus*	0/1	0/0	0/1	0/0	0/2
*Culiseta annulata*	0/18	0/2	1/101	0/62	1/183
*Culiseta longiareolata*	0/0	0/3	0/95	0/48	0/146
*Culiseta* sp.	0/0	0/1	0/1	0/0	0/2
*Culiseta subochrea*	0/0	0/0	0/1	0/0	0/1
*Ochlerotatus annulipes*			0/9	0/1	0/10
*Ochlerotatus berlandi*				0/1	0/1
*Ochlerotatus caspius*	0/519	1/594	1/953	3/804	5/2,870
*Ochlerotatus cinereus*	0/8	0/6	0/8	0/0	0/22
*Ochlerotatus communis*	0/0	0/0	0/16	0/12	0/28
*Ochlerotatus detritus*	0/9	0/2	1/15	0/21	1/47
*Ochlerotatus dorsalis*	0/1	0/0	0/0	0/0	0/1
*Ochlerotatus geniculatus*	0/7	0/5	0/8	0/2	0/22
*Ocherotatus punctor*	0/0	0/1	0/0	0/0	0/1
*Ochlerotatus rusticus*	0/0	0/0	0/1	0/0	0/1
*Ochlerotatus* sp.	0/4	0/0	0/34	0/45	0/83
*Uranotaenia unguiculata*	0/0	0/0	0/0	0/2	0/2
**Total**	**65/2,949**	**115/6,308**	**115/7,475**	**118/5,605**	**413/22,337**

### 2.2. Greece

Serosurveys in Greece conducted in human populations in 2007 showed that approx. 1% had antibodies to WNV. However, no human cases of WNV had been reported prior to 2010, when the virus emerged in the country and caused outbreaks for three consecutive years [46,47]. The causative strain was rapidly identified as belonging to WNV Lineage 2, and related sequences were obtained from *Cx. pipiens* mosquitoes collected by using CO_2_ traps in areas where human cases had been reported [48]. The genetic characterization of the whole genome revealed that the Greek strain (Nea Santa-Greece-2010) showed a close genetic relationship to the Lineage 2 strain that was detected in Hungary in 2004; an amino acid substitution H249P detected in the NS3 protein might be associated with increased virulence [33]. From 2010, a national surveillance plan was implemented, including testing of equines, reporting any positives to the World Organisation for Animal Health (OIE), sentinel chickens, doves and entomological testing. The aim of the entomological testing was first to map the mosquito species in the country, and secondly to test the collected mosquitoes for WNV.

WNV sequences containing the substitution H249P were obtained from wild birds and sentinel chickens [49,50], and also from *Culex* mosquitoes in subsequent years [51,52]. It was found that testing for seroconversion of sentinel chickens detected the presence of WNV one month earlier than the appearance of the first human cases [50]. Through the application of both generic flavivirus and WNV-specific primers for testing mosquito samples, a great number of insect-only flaviviruses have been detected in both *Culex* and *Aedes* spp. (unpublished observations). Although no association of these flaviviruses with public health has been observed, their involvement in the mosquito life-cycle and dual infection with pathogenic flaviviruses requires further investigation.

More than 60 mosquito species have been recorded in Greece, corresponding to seven genera: 23 species of *Aedes*, 14 *Anopheles*, 13 *Culex*, six *Culiseta*, two *Coquillettidia*, two *Orthopodomyia* and one species of *Uranotaenia* [53]. The dominant species during summer are: *Ae. caspius*, *Cx. pipiens* and *Cx. modestus* and three species of *Anopheles* (*An. sacharovi*, *An. pseudopictus* and *An. hyrcanus*). The first two species are present all over the country, whereas *Cx. modestus* and *Anopheles* spp. are found in wetlands, and especially in rice fields. With respect to the seasonal distribution of these dominant species, *Ae. caspius* is an early summer species, *Cx. pipiens* and *Cx. modestus* are found during the summer and the *Anopheles* spp. are detected during summer and early autumn. *Cx.* *pipiens* mosquitoes were tested at the locus CQ11 to distinguish between the two *Cx.* *pipiens* forms, pipiens and molestus, 71.4% were identified as *pipiens* (ornithophilic), 4.7% as *molestus* (anthropophilic) and 19% as *pipiens/molestus* hybrids (opportunistic biters), providing the first evidence that both *Cx.* *pipiens* biotypes are present in Greece, with a significant proportion being hybrids [50].

During an entomological survey in 2011 on behalf of the Hellenic Centre for Disease Control and Prevention, CO_2_ traps were deployed in 106 sites throughout Greece. On average the number of *Culex* spp. within 5 km from rice fields was 568 individuals per night per trap, while in peri-urban areas (sites adjacent to human settlements, not influenced by rice fields or wetlands) the respective number was 200. The analysis of extensive data sets from 20,000 hectares of rice fields in Thessaloniki, Northern Greece (10 stations, weekly landing rates at sunset, from June to September, 2006–2012, 848 samples in total), gave total average values of 14.8 individuals/15 min, representing 28.5% *Ae. caspius*, 21% *An. pseudopictus*, 19.5% *Cx. modestus*, 16.8% *Cx. pipiens*, 9.6% *An. hyrcanus* and 4.3% *An. sacharovi*. 

Due to its ability to transmit a range of arboviruses, there is currently an increasing concern in Greece for the spread of the invasive and anthropophilic mosquito *Ae. albopictus*, particularly in urban environments, after its first detection in 2004 in the northwestern region of the country [54]. In September 2008, *Ae. albopictus* was reported in a district of Athens, with increasing population abundance in subsequent years [55]. More recent data using oviposition trapping has confirmed its presence in settlements throughout Greece ([56] and *S. Mourelatos* unpublished data). 

### 2.3. Spain

WNV has been detected repeatedly in the Iberian Peninsula during the past ten years. Screening of mosquitoes for WNV detection in Spain has concentrated in wetlands in Western Andalucía (2001–2013) and Catalonia (2001–2009) [57,58,59]. Most of these activities have been instigated through research projects investigating various flaviviruses, and consequently the main objective was not early detection of potential WNV outbreaks but the characterization of flavivirus circulation in different areas of Spain. Screening of mosquitoes for flavivirus in Catalonia was stopped in 2010 due to the high costs of mosquito processing and molecular analyses [59]. A national WNV surveillance program was adopted in 2007. The plan involves surveillance in birds, horses and mosquitoes and considers different levels of surveillance. At the lowest level, attention is focussed on the detection of seroconversion in birds or detection of the virus in mosquitoes. When bird seroconversion is detected or extraordinary high mosquito abundance is observed, surveillance is initiated on seroconversion in horses and early detection of symptoms of disease. The last evaluation of WNV risk in Spain explicitly recommended support for surveillance in horses and mosquitoes, due to its usefulness in detecting WNV circulation in advance of the appearance of cases in humans. However, no regular activities to detect WNV in mosquitoes related to the surveillance plan are currently undertaken and mosquito monitoring depends largely on the existence of research projects funded by Regional, National or European research agencies. A national plan of entomological surveillance in Spanish harbours and airports has been conducted since 2008 and coordinated by the University of Zaragoza. The main objectives of the program are: (1) detection of imported vector species that may be competent for the transmission of infectious diseases and (2) monitoring the potential expansion of new introductions of *Ae. albopictus*. Entomological surveillance focuses both on adult mosquitoes and prospecting for areas of larval presence, particularly for exotic species. Unfortunately, this scheme does not support testing for flaviviruses in collected samples

Monitoring of mosquitoes in Spain has enabled the detection of WNV and USUV years before the occurrence of cases in humans or horses [58,60] and the description of new viruses and WNV lineages [8]. The analysis protocols have been similar in all the cases. Females were captured with CO_2_ baited traps (CDC and BG traps), classified to species level and analysed in monospecific pools of up to 50 females using flavivirus generic primers. A fragment of NS5 gene was sequenced in positive pools.

Overall more than 218,507 mosquitoes, separated into 12,844 pools from 22 species have been screened (Table 4). WNV positive pools have been detected in *Cx. pipiens* in 2006 and *Cx. perexiguus* in 2008 [60]. USUV has been detected both in Catalonia in 2006 [58] and in Andalucía in 2009 [60] in *Cx. pipiens* and *Cx. perexiguus*, respectively. Overall, eight out of 527 *Cx. perexiguus* pools tested positive for WNV or USUV (1.5%) and two out of 3,763 *Cx. pipiens* pools were positive for these viruses (0.05%). Additionally a novel mosquito-borne flavivirus has been repeatedly detected in *Oc. caspius* in Andalucía, but it is still unknown if this virus is able to infect vertebrate cells. Finally, two mosquito-only flaviviruses have been reported in Spain [61,62].

**Table 4:** Number of pools and female mosquitoes from each species tested in Spain, based on published studies (see text).

Mosquito species	Pools	Mosquitoes	WNV + pools	USUV + pools
*Aedes albopictus*	28	62	-	-
*Aedes vexans*	42	433	-	-
*Anopheles algeriensis*	59	241	-	-
*Anopheles atroparvus*	644	6,520	-	-
*Anopheles claviger*	2	2	-	-
*Anopheles hyrcanus*	1	1	-	-
*Anopheles maculipennis*	1	236	-	-
*Anopheles plumbeus*	5	12	-	-
*Anopheles* sp.	9	89	-	-
*Coquillettidia richiardii*	62	147	-	-
*Culex modestus*	1,181	21,426	-	-
*Culex perexiguus*	527	7,366	7	1
*Culex pipiens*	3,763	55,469	1	1
*Culex* sp.	69	551	-	-
*Culex theileri*	1,413	37,512	-	-
*Culiseta annulata*	114	212	-	-
*Culiseta longiareolata*	340	851	-	-
*Culiseta subochrea*	17	691	-	-
*Culiseta* sp.	4	4	-	-
*Ochlerotatus berlandi*	2	2	-	-
*Ochlerotatus caspius*	3,621	83,651	-	-
*Ochlerotatus detritus*	486	2,998	-	-
*Ochlerotatus geniculatus*	4	13	-	-
*Ochlerotatus pulcritarsis*	4	5	-	-
*Ochlerotatus* sp.	2	3	-	-
*Uranotaenia unguiculata*	7	8	-	-
Species not reported	437	2	-	-
**Total**	**12,844**	**218,507**	**8**	**2**

Sequence data derived from WNV isolations in Spain have demonstrated that two different lineages circulate, Lineage 1 and a new lineage currently only detected in Spain [60]. Sequences from WNV Lineage 1 in birds in 2007 and in mosquitoes in 2008 had high genomic homology (>99.5%), suggesting that WNV over-wintered in the area at least between these seasons, and probably in subsequent years [63]. Mosquito monitoring has provided a basic understanding of the dynamics of WNV amplification and transmission risk to different species [23], however, more information is needed to estimate the seasonal and geographic risk of WNV outbreaks.

### 2.4. Switzerland

WNV has never been detected in mosquitoes in Switzerland, but due to positive findings in northern Italy surveillance activity is being established for WNV and other relevant mosquito-borne viruses. Initially, this was introduced in southern regions but with the long term aim of implementing a broad mosquito surveillance system across the country. The scope of the surveillance activity was defined based on a risk assessment performed for selected mosquito-transmitted viruses considering their emergence in Europe and prevalence worldwide, the availability of competent vectors in Switzerland and the potential impact of an outbreak on public health. Accordingly, WNV, Dengue virus (DENV), Chikungunya virus (CHIKV) and USUV were rated as being of prime interest, but several other mosquito-transmitted bunya- and alphaviruses were also included in the surveillance project.

**Figure 2:**
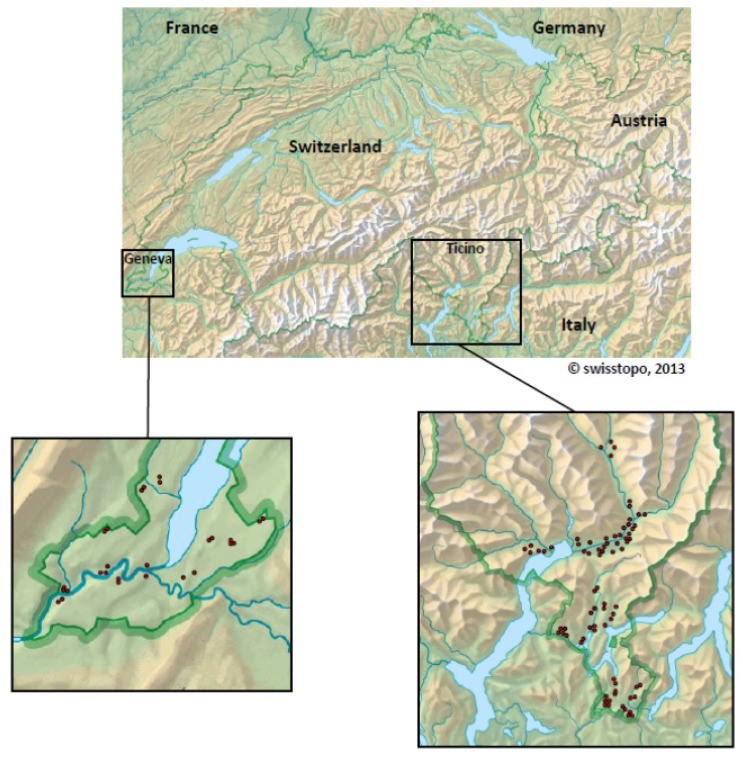
Map of the regions of Switzerland enrolled in the mosquito surveillance programme in 2012. Sampling sites are indicated by red points in the lower panels.

Methods for the molecular detection of viruses in mosquitoes were established by adapting a published virus extraction method for ticks to the different mosquito species [64] and by optimising in-house and published protocols for quantitative RT-PCR and generic RT-PCR. Capture methods, transport logistics, mosquito identification, and analysis were introduced within the framework of a pilot study performed in 2010 in the canton of Ticino. Methods were further optimised in 2011. In 2012 the number of collected mosquitoes was increased and the areas covered by the surveillance programme expanded to include areas in the canton of Geneva in the western part of Switzerland (Figure 2).

In 2011/2012, a total number of 16,982 *Culex* mosquitoes (mainly *Cx. pipiens*), 4,597 *Ae. vexans*, 3,938 *Ae. albopictus* and a significant number of other mosquito species were collected in the canton of Ticino and Geneva (Table 5). In Ticino, *Culex* species were caught overnight using gravid traps at 42 different sites representing six regions of natural and inhabited areas. *Ae. vexans* were trapped at six sites, mainly in natural areas using CDC traps, and *Ae. albopictus* were trapped at 60 sites in four inhabited areas using either a vacuum device or by hand. Collected mosquitoes were immediately frozen and sorted under frozen conditions. RNA extraction from mosquito pools was performed using a protocol optimised for mosquitoes and extraction-efficiency was monitored in all samples by applying a Mengo-virus as an external extraction and PCR control. The molecular analysis of all mosquito pools revealed that none of the *Culex* or *Aedes* mosquitoes collected in 2011 and 2012 were positive for WNV and none of the *Ae. albopictus* pools was positive for DENV or CHIKV. In several of the *Culex* mosquito pools sequences of USUV were detected and the presence of the virus could be confirmed by virus-specific RT-PCR and subsequent sequencing. The application of the generic nested RT-PCRs to all mosquito pools allowed the identification of mosquito-specific flaviviruses of unknown relevance for human or animals in several of the *Culex* and *Aedes* mosquito pools. Although no human pathogens were detected, the results from this study proved that the methods applied are suitable for the detection of flaviviruses in mosquitoes. Attempts are underway to expand the surveillance to other regions of Switzerland in order to achieve a more representative coverage of the country. 

**Table 5:** Mosquito species collected in Switzerland, in the canton of Ticino and Geneva, in the period 2011–2012. Indicated are the numbers of collected mosquitoes and the numbers of positive pools. (-) indicates where no mosquitoes of corresponding species were found and (--) where mosquitoes were not analysed.

Mosquito species	Mosquitoes		WNV+ pools	Usutu+ pools	Mosquito-Flaviviruses
	Ticino	Geneva			
*Aedes albopictus*	3,938	-	0	0	0
*Aedes cinereus/geminus*	1,791	1	--	--	--
*Aedes vexans*	4,597	-	0	0	33
*Aedes* sp.	1	1	--	--	--
*Anopheles claviger*	2	31	--	--	--
*Anopheles plumbeus*	17	2	--	--	--
*Anopheles maculipennis*	349	28	--	--	--
*Coquillettidia buxtoni*	2	-	--	--	--
*Coquillettidia richiardii*	53	46	--	--	--
*Culex hortensis*	9	-	--	--	--
*Culex modestus*	-	-	--		--
*Culex pipiens/* *torrentium*	12,780	2,129	0	41	3
*Culex* sp.	2,061	12	0	0	0
*Culiseta annulata*	40	14	--	--	--
*Culiseta* sp.	1	6	--	--	--
*Ochlerotatus cantans*	299	5	--	--	--
*Ochlerotatus caspius*	5	1	--	--	--
*Ochlerotatus geniculatus*	9	1	--	--	--
*Ochlerotatus sticticus*	5,654	1	--	--	--
**Total**	**31,608**	**2,278**	**0**	**41**	**36**

Thirty-eight mosquito species are known in Switzerland, including the two invasive species *Ae. albopictus* in the most southerly part of the country and *Ae. japonicus* in areas north of the Alps. However, little is known about the distribution of the mosquitoes or their seasonal abundances. In order to contribute towards devising a risk assessment of WNV transmission in Switzerland, a research project has been launched in 2012 aimed at determining the vector capacity traits of Swiss mosquito populations. Species richness and seasonal abundances of mosquitoes are determined by regularly collecting eggs, larvae, pupae and adults in two natural zones adjacent to extended wetlands (as putative sites of virus introduction by migratory birds) and two suburban sites (as putative sites of virus transmission to humans) on either side of the Alpine crest over three consecutive years. Host preferences of the mosquitoes are determined by using animal-baited traps, and the vector competence of abundant Swiss mosquito populations for WNV is investigated under laboratory conditions. These experiments are performed under different, realistic environmental conditions (spring/autumn and summer fluctuating temperature regime) as it was recently shown that such daily temperature fluctuations influence the vector competence [65,66]. In addition, vector competence is also investigated for Sindbis (=Ockelbo) virus which is endemic in the EU and which, as a virus adapted to cooler climate, also serves as positive control in the transmission experiments. 

Special attention is devoted to the invasive species *Ae. japonicus* which has become the most abundant species at suburban sites north of the Alps and which is rapidly spreading [67,68,69]. This species, which earlier had been introduced into the USA, could play a role as bridge vector as it readily feeds on birds and mammals, including humans, and it has been shown to be a competent laboratory vector of several arboviruses, including WNV [70], DENV and CHIKV [71]. In Switzerland, *Ae. albopictus* was recorded for the first time in summer 2003, in the southern part of the country near the Italian border [72] within a monitoring program started in 2000 by the local Mosquito Working Group (Gruppo Lavoro Zanzare, Canton of Ticino). This species became established in 2007 and started to expand northwards [73]. In 2012, a total of 50 communities were infested by *Ae.* *albopictus* and its density reached levels at which an autochthonous transmission of DENV or CHIKV from an infected person coming from endemic countries cannot be excluded.

### 2.5. United Kingdom

WNV has never been detected in the United Kingdom (UK), although a single paper has reported detection of seropositive wild birds [74]. The results were never repeated in wildlife and there have been no human or equine cases in the UK. Mosquito surveillance in the UK has been conducted by the Medical Entomology group at Public Health England (formerly the Health Protection Agency) since 2005. Over the past eight years these activities have taken many forms, both passive and active surveillance, but essentially have been targeted towards WNV mosquito vectors and invasive mosquito species. Thirty-four mosquito species have been reported in the UK [38,75]. In 2005, a passive mosquito surveillance scheme was established encouraging both the public and environmental health pest control departments to submit samples for identification. This provided a forum for suspect invasive *Aedes* species to be identified [76]. Historical records of UK mosquito distribution were also collected, and the combination of this data is published by the UK National Biodiversity Network [77]. A number of peer-reviewed review and geospatial risk mapping papers were published which identified key potential UK-specific WNV enzootic and bridge vectors [78], potential mosquito vector species of other EU arboviruses [38], new mosquitoes species to the UK [79,80] and a risk map of potential sites for *Ae. albopictus* establishment and seasonal activity [81].

In 2010, active mosquito surveillance was conducted at 11 UK sea- and air-ports with a view to training port health officers and establishing cost-effective sustainable mosquito surveillance for exotic mosquitoes arriving through ports [82,83]. Key putative WNV vector species (identified through UK specific ecological reviews) were targeted at an additional 10 nature reserves across England. Unusual findings were followed up through targeted field surveys. This included the finding of significant populations of *Cx. modestus* in the North Kent marshes [80] with additional specimens found in Cambridgeshire [79]. Unusual records of *Oc. sticticus* and *Ae. vexans* were also identified, with subsequent follow up studies. Standard sampling techniques at nature reserves, ports, and during additional wetland management studies [84,85] have included Mosquito Magnet traps, larval sampling, oviposition traps and BG sentinels. During 2010 and in subsequent years, mosquito magnets were run for four nights every two weeks between April and October. A total of ~22,000 mosquitoes were collected during 2010, comprising of 18 species. One thousand specimens were extracted from the nationwide and Cambridgeshire wetland studies for pathogen analysis by AHVLA of small pools of five individuals, with the identification of a mosquito-only flavivirus [62].

In addition, surveillance for invasive *Aedes* species has been conducted at a number of imported tyre companies across England, targeting those companies with the greatest trade in tyres with the EU, far-East and North America. Sampling strategies included larval sampling of tyres, oviposition traps and adult sampling using BG sentinels. So far no invasive *Aedes* have been found in the UK.

### 2.6. Germany

**Figure 3:**
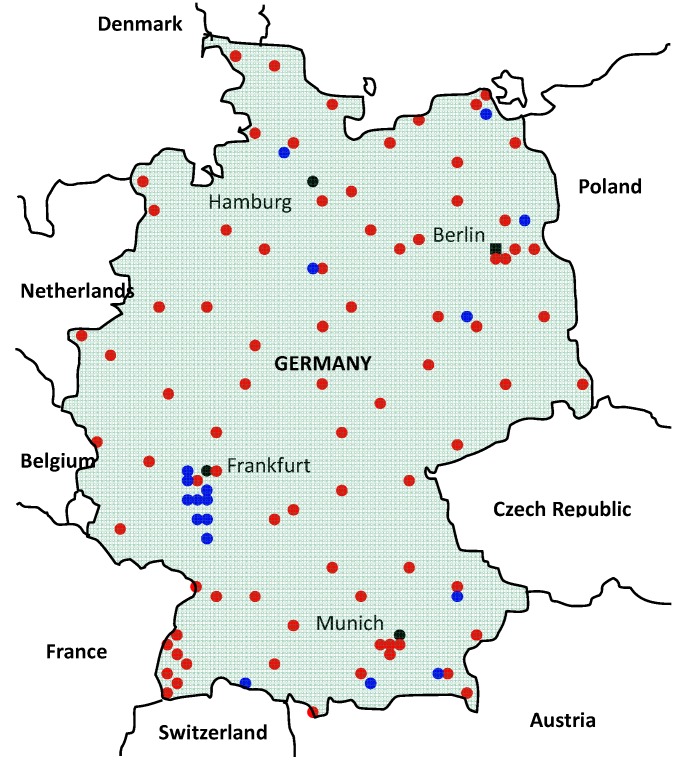
Map showing the locations of stationary mosquito traps within Germany. Traps operated by the German FLI/ZALF consortium are indicated in red, traps operated by the KABS/BNI consortium are indicated in blue (see text for details).

Four different mosquito-borne viruses which are pathogenic for vertebrates have been found in Germany to date: Tahyna virus (TAHV), Sindbis virus (SINV), Batai virus (BATV) and USUV. In 1968, Tahyna virus (TAHV) was isolated from mosquitoes that were trapped around Baunach in Bavaria [86]. TAHV is the causative agent of Valtice fever, an influenza-like illness occurring in summer and early autumn. Since these early discoveries, virus surveillance in mosquitoes, humans and animals was not carried out regularly and therefore, longitudinal data sets are missing. Germany is currently considered to be free of WNV infections. Extensive surveillance studies on thousands of wild and domestic birds and of equines [87,88] as well as on mosquitoes have been carried out to substantiate this assumption as far as possible. However, given the endemic circulation of WNV in some parts of Italy, in Hungary and in other Balkan countries as well as in Southern France, an incursion by global trade, travel and natural spread is possible. Therefore, extensive surveillance activities for WNV in particular and for other arboviruses have been carried out since 2007. This includes studies to map the presence and distribution of relevant vector species (mosquitoes, biting midges *etc*.) as well as of the arboviruses carried by them [89]. These surveillance studies are carried out by two large research consortia which collaborate to a certain degree. The first consortium works nationwide and is operated by the Friedrich-Loeffler-Insitute (FLI) and the Leibniz Centre for Agricultural Landscape Research (ZALF), while the second is more focused on Southern Germany and involves scientists from the Kommunale Aktionsgemeinschaft zur Bekämpfung der Schnakenplage e.V. (KABS) [90] and the Bernhard Nocht Institute (BNI). At approximately 120 sites (Figure 3), both groups operate up to four different types of traps (BG sentinel CO_2_ trap, EVS trap, gravid trap, ovitrap) in natural wetlands, in urban areas and at other ecologically or epidemiologically relevant locations, such as islands, brackish water areas, airports, train stations, cemeteries and zoological gardens in order to collect a wide range of species in their particular habitats. Special attention was also given to potential entry routes for newly invasive species particularly along highways in the Upper Rhine valley and towards the southern borders to Austria and Switzerland. Captured mosquitoes are dried and identified to species morphologically or genetically. Among several tens of thousands of mosquitoes have been processed, several mosquito species new to Germany, such as specimens of *Ae. albopictus*, *Ae. japonicus*, *An. daciae* and *Cs. longiareolata* were reported [91,92]. Moreover, mosquito pool samples were examined by PCR for the presence of flavi-, alpha- and bunyaviruses. To allow a better comparison many of the real-time RT-PCRs which are run at FLI and BNI have been shared between both institutions. 

To date, more than 150,000 mosquitoes have been assayed for the presence of viruses. In 2009, SINV was isolated from *Culex* and *Anopheles* mosquitoes belonging to the Maculipennis complex that were exclusively trapped in the city of Weinheim, south-west Germany [93]. SINV is the causative agent of a febrile illness in humans associated with maculopapular rash and joint pain. In 2009, BATV was also isolated from *Anopheles maculipennis* sensu lato mosquitoes trapped around the village of Waghäusel [94]. BATV may cause a mild illness among sheep and cattle. Thus, 195 serum samples from cattle around the village of Waghäusel were investigated for BATV-specific-IgG antibodies and two samples were tested positive, demonstrating past BATV infections. In 2010, USUV was isolated from *Cx. pipiens* mosquitoes trapped in the city of Weinheim [95]. Since June 2011, considerable mortality in wild and captive bird species was observed in south-west Germany. Consequently, 168 dead birds were tested for USUV and USUV RNA was detected in 80 individuals from six species. Thus, the mortality of birds was shown to be associated with the emergence of USUV [96]. 

## 3. Discussion

The threat of WNV can be mitigated through the early detection of virus within mosquito populations. Mitigating actions include vector reduction through application of larvicides, vaccination of equids and increasing awareness amongst the public including measures to reduce mosquito biting rates. Early detection through surveillance can take various forms including virus detection, seroconversion and overt disease, and in many different populations including mosquito vector species, avian host, susceptible/non-susceptible mammals and humans. With such a range of surveillance options available, the search for the most effective approach within the budgetary constraints of any one country is continuous [97]. In this article we have focused on the varied approaches taken by a small sample of countries across Europe to deliver entomological surveillance for WNV. Surveillance data from the countries included in this survey show that WNV has been detected in mosquitoes in Italy, Spain and Greece but not in Switzerland, Germany and the UK. The species most commonly infected was *Cx. pipiens*, but WNV was also detected in *Cx. modestus*, *Oc. caspius* and *Cx. perexiguus*. This complements similar findings in other European countries [98]. The reasons for this distribution are likely to be linked to climatic conditions that favour vector abundance as seen in the numbers of mosquitoes sampled in Italy and Greece that exceed the numbers observed in countries to the north. It also reflects possible points of WNV entry into Europe through bird migration.

Evidence from Italy and Spain suggests that its detection in mosquitoes precedes the appearance of human or equine cases arguing for its application in any surveillance strategy for WNV and other zoonotic arboviruses in areas at risk of incursion. Data collected in Greece in the course of the annual WNV outbreaks observed since 2010 demonstrate that surveillance activities obtain information on the relevant vectors involved in transmission and on the circulating virus strain, thus providing important information for preventing virus transmission and the diagnostic procedures needed. Continuous entomological surveillance also enables improved estimation of the risk of WNV transmission in different areas during any particular year; allowing evolution of preventive control measures for the human population in response to data on vector activity and virus detection. 

In countries with no prior evidence for the presence of WNV, the extent of surveillance needs to be modified in response to developments in those that are adjacent in order to avoid unnecessary testing. For example, the introduction of mosquito surveillance in Germany and Switzerland was a direct response to the emergence of WNV in Italy. Even within countries, a high degree of cooperation between specialists (entomologists, virologists, risk analysts, public health professionals) is required to deliver an effective surveillance programme. Achieving this across national borders presents an additional challenge. Such supra-regional or supra-national level cooperation requires considerable coordination of methodologies to achieve meaningful comparison of data and a willingness to share information freely. Agreements between research groups and their funding bodies from individual European countries would be integral to achieving a Europe-wide surveillance network. Coordinating trapping procedures, the numbers and species of mosquitoes collected, and the molecular protocols applied for analysis would be a desirable objective. From the data obtained from the countries where WNV was detected, *Cx. pipiens* is most commonly associated with the virus in Europe and surveillance should focus on sampling this species. Countries which were successful in identifying relevant viruses in mosquitoes could support others by providing test material, including positive controls, to test their protocols. Furthermore, the network should aim to offer continuous improvements in the methodologies used for the capture, identification and analysis of mosquitoes and test new approaches. The location of surveillance sites would be critical to this and risk analysis of areas that are likely to be foci of WNV introduction, particularly large wetland areas and adjacent equine and human populations, are needed across Europe. 

Another critical aspect of entomological surveillance is achieving rapid species identification, pooling and virus testing. This needs to be achieved within a worthwhile timeframe (weeks) to generate results that can be passed on to reactive public health and veterinary authorities. This presents a series of challenges. Firstly, accurate identification of specimens is needed by those with sufficient experience to positively identify *Culex* species. Molecular identification of closely related species within the *Cx. pipiens* complex is possible [99], however its application may be prohibitive in terms of cost and time if applied to individual mosquitoes. Pool sizes need to be defined and testing methodologies need to be validated. In this again, a network of countries would assist in spreading best practise and maintaining standards through coordinated ring-trials. High volume testing has been achieved in most countries through the use of pan-flavivirus and WNV-specific RT-PCR. Although the detailed methodologies are not presented in this report, they can be found within cited publications. Most laboratories screen with a generic-flavivirus PCR (see ref. [100] for examples of generic-flavivirus assays). This provides a breadth of virus targets and has led to the discovery of a wide variety of insect-only flaviviruses in numerous mosquito populations across Europe [61,62,101]. USUV has also been detected in many European countries and is expanding its range [96]. Their presence necessitates further testing, and cost, to confirm the virus species detected. A number of countries sampled in this study survey for other mosquito-borne viruses including SINV and BATV.

A useful by-product of mosquito surveillance is the generation of abundance and population data on indigenous mosquito species and the detection or spread of invasive species such as *Ae. albopictus* and *Ae. japonicus*. This data is of particular importance in assessing the ability of WNV and other arboviruses/mosquito borne pathogens such as DENV and malaria to establish in Europe, at a regional level.

## 4. Conclusions

The extent of surveillance for WNV varies widely between European countries ranging from large multi-faceted programmes that include mosquito sampling, to *ad hoc* testing of samples through small-scale research projects. In order to coordinate surveillance between countries, each country should have a surveillance plan, including those activities that maximise detection of the virus with the express purpose of providing data on threats to human and equine health. In developing this for WNV surveillance there is a clear need to incorporate statistical and risk analysis to survey design to optimise the collection strategy and location respectively. This has been applied in a number of the countries considered in this review. Surveillance for WNV within European mosquitoes has been implemented in most countries across Europe and has successfully detected the virus in a small number of species in countries around the Mediterranean Basin but at very low prevalence. Coordination of methodologies, including trapping protocols (trap type, location, frequency of sampling), species identification and virus testing, would greatly assist in data comparison and sharing, and should be a goal for supra-regional surveillance for future incursions of WNV. 
